# A rare complication of gallstones ended with spontaneous cholecystocutaneus fistula in an old man: A case report

**DOI:** 10.1016/j.ijscr.2020.01.008

**Published:** 2020-01-23

**Authors:** Kusay Ayoub, Mohamad shadi Alkarrash, Mohammad Nour Shashaa, Aya Zazo, Roaa Rhayim, Nihad Mahli

**Affiliations:** a Surgery Department, Faculty of Medicine, Aleppo University Hospital, University of Aleppo, Aleppo, Syria; b Faculty of Medicine, University of Aleppo, Aleppo, Syria

**Keywords:** Cholecystocutaneous, Fistula, Abscess, Gallstone, Cholelithiasis, Spontaneous, fistula

## Abstract

**Introduction:**

Cholecystocutaneous fistula is an extremely rare complication of gallstones. The majority of the fistulae localize into the right upper quadrant of the abdomen. Diagnosis is not easy because the symptoms are nonspecific. External biliary fistula is more common in females between the 5th-7th decades because in this age group the frequency of cholecystitis is increased.

**Case presentation:**

A 65-year-old man presented with a swelling in the right hypochondrium. He had not complained of any serious symptoms. Laboratory investigations were normal. Both abdominal ultrasound and Multislice computed tomography (MSCT) showed that the gallbladder contained stones, in addition to a cystic mass in the abdominal wall. The previous findings suggested that there was an abscess. So, the patient underwent laparoscopic procedure, and we found a fistula between the gallbladder and the abdominal wall causing the abscess. The greater omentum was adherent to the inflamed gallbladder. So that we transformed into open procedure to perform cholecystectomy and resect the fistula.

**Discussion:**

Biliary fistulae have two types, external and internal. The most common type is internal. External biliary fistulae emerge like a complication of cholecystolithiasis in general. Spontaneous cholecystocutaneous fistulae occur due to acute inflammation caused by cholecystitis or chronic gallstones disease. The most common clinical manifestation is an abscess in the abdominal wall that discharges bile to the outside.

**Conclusion:**

Computed tomography is considered the gold standard in diagnosing a spontaneous cholecystocutaneous fistula, and it should be considered as a differential diagnosis for any abscess that is evident in the abdominal wall.

## Highlights

• Cholecystocutaneous fistula is an extremely rare complication for gallstones.
• The diagnosis is not easy because symptoms are nonspecific.
• Computed tomography considers the gold standard in diagnosing this case.
• First procedure was laparoscopic, but greater omentum adherent to the gallbladder.
• So we turned to open technique to perform cholecystectomy and resection the fistula.

## 1 Introduction

Spontaneous cholecystocutaneous fistula is a rare connection between gallbladder and skin. Thelisus was the first one who reported cholecystocutaneous fistula in 1670. Then, 169 cases of biliary fistula in the 19th century defined by Courvoisier [1]. The first case of spontaneous external biliary fistula that gallstones were not complicating factor reported in 1991 [2]. The majority of fistulae are internal (duodenum 77%, colon 15%), while external fistulae are unusual [3]. External biliary fistula is more common in females between the 5th-7th decades because, in these decades, the frequency of cholecystitis increased. Most of these fistulae emerge into the right upper quadrant (48%) or umbilicus (27%) [4]. Nonspecific symptoms of this rare complication of gallbladder disease make the diagnosis a real challenge in medicine [5]. We report an unusual case of cholecystocutaneous fistula revealed by an abscess in the right hypochondriac area in an old-man. This work is reported accord to the SCARE criteria [6].

## 2 Case presentation

**Fig. 1:**
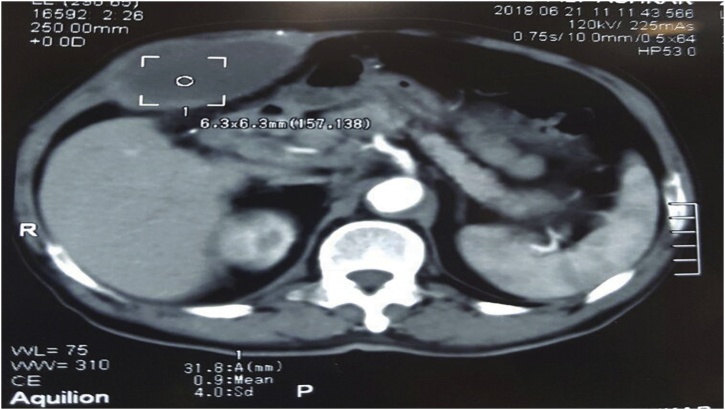
CT scan showing the cystic mass in the abdominal wall.

**Fig. 2:**
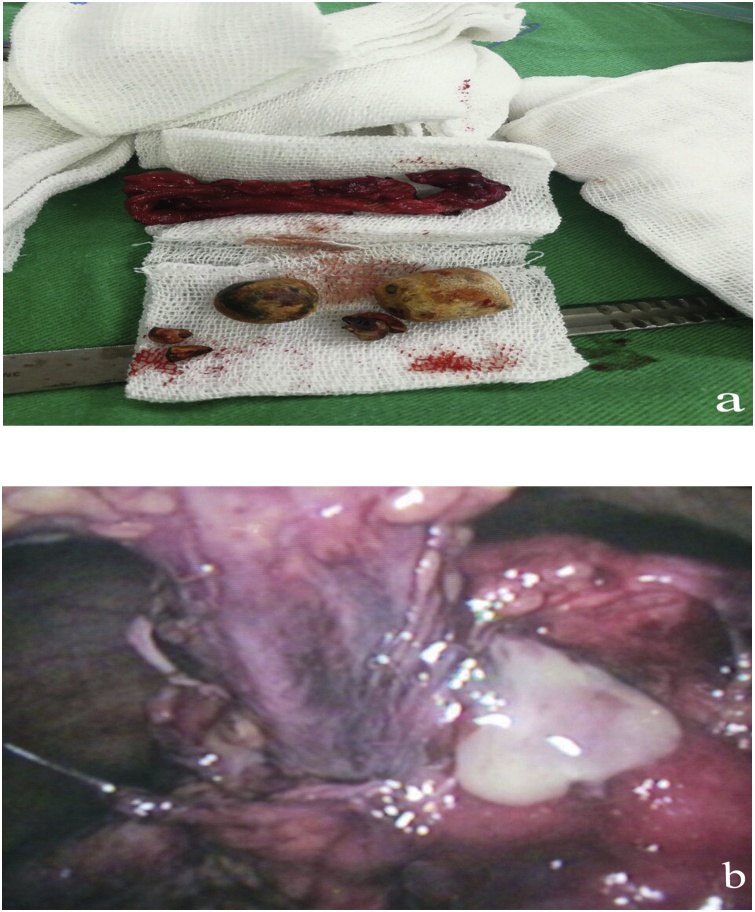
a The resected gallbladder, which was contained multiple stones. b Fistula between the gallbladder and the abdominal wall during laparoscopy.

A 65-year-old man presented with a complaint of a swelling in the right hypochondriac area. There was no vomiting or nausea, no changes in the weight, no hyperthermia, but the patient was a smoker and had hypertension. Physical examination showed slight localized tenderness, no redness was noticed, and the rest of the abdominal examination was normal. Weinberg’s test was negative. Blood tests revealed a white blood cell count of 8800/mm^3^(within normal), lipase 17 U/l, ALT of 0.7 U/L, AST of 23 U/L. Abdominal ultrasound showed a cystic mass in the abdominal wall and thickened gallbladder containing stones; on the other hand, common bile duct (CBD), liver, spleen, kidneys were all within normal. Multislice computed tomography (MSCT) showed the cystic mass in the abdominal wall (4.6 × 7.8) cm [Fig. 1]. Then, the previous findings suggested that the cystic mass was an abscess, and drainage was done. Pharmacological therapy and antibiotics were given to the patient. Because of gallstones, there was an indication to go into surgery, which was performed under general anesthesia, and the patient underwent laparoscopic investigations. During the surgery, there was severe edema in the gallbladder, a fistula between the gallbladder fundus and the abdominal wall led to an abscess in the abdominal wall, and the greater omentum was adherent to the inflamed gallbladder. That is why we were not able to do the cholecystectomy. Therefore, the patient was transformed into an open technique; and cholecystectomy and resection of the fistula were done [Fig. 2a and b]. The patient was followed for three months after the surgery, and the radiographic and laboratory tests were all within normal.

## 3 Discussion

Biliary fistulae have two types internal and external. Internal fistulae are more common than external fistulae, considering that 75% of internal fistulae connect with the duodenum, 15% connect with the colon, and 10% connect with the stomach or jejunum or have a connection with many organs [5].

External biliary fistulae usually occur as a complication of cholecystolithiasis. However, external biliary fistulae can occur secondary to cholangiocarcinoma and biliary laceration during the surgical procedure [5,7]. Spontaneous cholecystocutaneous fistula is rarely reported in the medical literature. The first description of this disease was by Thilesus in 1670. Spontaneous cholecystocutaneous fistula usually affects women over the age of 60 [8]. This disease occurs due to acute inflammation caused by cholecystitis or chronic gallstones disease. However, some cases in medical literature described spontaneous cholecystocutaneous without gallstones [9]. The blockage of the cystic duct causes high pressure inside the gallbladder, which compromises blood circulation and causes necrosis in the gallbladder wall. The fistula was formed with adjacent areas involving the abdominal wall [10]. Cholecystocutaneous fistula usually opens in the right hypochondrium but can open in many other sites involving right iliac fossa, left hypochondrium, the umbilicus, and right gluteal region [10,11]. The most common clinical manifestation is an abscess in the abdominal wall that discharges bile to the outside [10]. There are several differential diagnoses for this disease including epidermal cyst, discharging tuberculosis, pyoderma granuloma, chronic osteomyelitis of the ribs, and metastatic cancer [12]. Investigation usually starts with an abdominal ultrasound, which is useful for assessing the gallbladder. Computed tomography (CT) is considered the best investigation in the diagnosis. CT fistulogram confirms the diagnosis where the fistula appears connected with the gallbladder [10,12]. Routine blood tests are useful when there is an abscess, and the general condition of the patient is poor [10]. Endoscopic retrograde cholangiopancreatography (ERCP) should be performed if there are gallstones in the main bile duct [8,13]. Treatment of spontaneous cholecsytocutaneous fistula starts with drainage of any abscess and pharmacological therapy with antibiotics. Cholecystectomy with removal of the fistulae tract is the definitive management [10]. The first case was treated with the laparoscopic technique by Kumar in 1998 [14]. In general, CT is considered the best procedure in diagnosing a spontaneous cholecystocutaneous fistula. Eventually, the spontaneous cholecystocutaneous fistula should be considered as a differential diagnosis for any abscess in the abdominal wall.

## 4 Conclusion

The spontaneous cholecystocutaneous fistula is rarely reported in the medical literature. Fistulae can form in any elderly patient who underwent previous percutaneous treatment for acute cholecystitis and had recurrent cholecystitis. CT is considered the best investigation in the diagnosis. Treatment includes surgical and pharmacological therapy. This disease should be considered as a differential diagnosis for any abscess in the abdominal wall.

## Sources of funding

There are no sources of funding.

## Ethical approval

Not required for case reports at our hospital. Single case reports are exempt from ethical approval in our institution.

## Consent

Written informed consent was obtained from the patient for publication of this case report and accompanying images. A copy of the written consent is available for review by the Editor-in-Chief of this journal on request.

## Author contribution

Kusay Ayoub: managed the patient and did the surgery, design of the study, data interpretation and analysis, revision.

Mohamad shadi Alkarrash: data collection, revising critically, wrote the manuscript.

Mohammad Nour Shashaa: review and editing, data analysis, wrote the manuscript.

Aya Zazo: patient care, revision, corresponding author.

Roaa Rhayim: design of the study, revision, Validation.

Nihad Mahli: the supervisor, patient care, revising critically.

All authors read and approved the final manuscript.

## Registration of research studies

NA.

## Guarantor

Dr.Kusay Ayoub.

## Provenance and peer review

Not commissioned, externally peer-reviewed.

## Declaration of Competing Interest

The authors declare that they have no conflict of interest.
